# Impact of Compaction Parameters and Techniques on MUPS Tablets

**DOI:** 10.3390/pharmaceutics17101347

**Published:** 2025-10-18

**Authors:** Daniel Robin Thio, Paul Wan Sia Heng, Lai Wah Chan

**Affiliations:** GEA-NUS Pharmaceutical Processing Research Laboratory, Department of Pharmacy and Pharmaceutical Sciences, National University of Singapore, 18 Science Drive 4, Singapore 117543, Singapore; daniel.thio@u.nus.edu (D.R.T.); paulwsheng@outlook.com (P.W.S.H.)

**Keywords:** pellet coat damage, multi-unit pellet system (MUPS) tablet, precompression, trilayering, tableting rate, pellet lubrication

## Abstract

**Background/Objectives:** Compaction of sustained release coated pellets into tablets is associated with damage to the functional coat and loss in sustained release. The influences of precompression, trilayering, and tableting rate on the compaction of sustained release coated pellets into tablets are not well defined and were herein investigated to enhance the current limited understanding of these factors. Methods: Pellets coated with acrylic polymer (AC) or ethylcellulose (EC) were combined with filler material and compacted into multi-unit pellet system (MUPS) tablets prepared using different levels of precompression, as a trilayered MUPS tablet and at different tableting rates. The physical properties of the resulting MUPS tablets were evaluated. Trilayering was achieved by adding cushioning layers at the top and bottom of the MUPS tablet to avoid direct contact of pellets with punch surfaces. **Results:** With precompression, slightly stronger MUPS tablets were made compared to the tablets without precompression for EC pellets but not AC pellets. However, precompression led to a slight reduction in pellet coat damage for AC pellets but not EC pellets. Trilayering led to significant reductions in pellet coat damage and significant increases in tablet tensile strength. When EC pellets were lubricated with sodium stearyl fumarate, pellet coat damage was significantly lower. Increasing the tableting rate from 20 to 100 rpm did not result in increased pellet coat damage but in significantly weaker tablets due to the shorter dwell time. **Conclusions**: This study provides key insights on how compaction parameters and techniques could be altered to produce better MUPS tablets.

## 1. Introduction

Sustained release dosage forms provide a variety of benefits to drug therapeutics. These benefits include reduced dosing frequency [1], prolonged therapeutic activity [2], and lower incidence of side effects [2]. Formulators can create sustained release products in the form of multi-unit pellet system (MUPS) tablets, in which drug-loaded pellets coated with a sustained release polymer are compacted with filler material [3,4,5,6,7,8,9,10,11,12,13]. Once ingested, the MUPS tablet disintegrates, releasing its payload of coated pellets and sustained drug release begins [14]. There are many benefits to using this approach over coated single-unit tablets or capsules, such as the increased area for drug absorption [15,16], independence from gastric emptying [17,18], possibility of subdividing the dosage form, and lower risk of drug dose dumping or blockage due to lodged dosage form in the gastrointestinal tract [17,18]. Some MUPS tablet products are commercially available [3,11]. Recent studies have evaluated various materials for use in MUPS tablets, ranging from the filler material [4,7,9,11] to pellets [10,11,12,19]. There has also been some interest in the compaction parameters, which will be expounded upon below.

Precompression and tableting rate play an important role in determining the quality of the tablets produced, particularly when it pertains to capping and productivity [20,21,22,23,24,25,26]. In the tablet compaction cycle, the precompression which occurs just before the main compaction cycle has the main objective of removing air between the tablet components to allow the formation of a stronger tablet during the main compaction [22,24,25,27]. It has also been reported that precompression increases the overall duration of compaction, which helps to increase the time-dependent plastic deformation of some materials such as microcrystalline cellulose (MCC) to form stronger tablets [21,28,29,30,31]. Although precompression has been associated with the formation of stronger tablets, the benefits derived depend on the type of components as some formulations do not benefit from the precompression. To date, the benefit of precompression for MUPS tablets containing sustained release pellets is unclear as it has not been clearly established in the literature.

The tableting rate is a highly important parameter in the manufacture of tablets because it directly influences the tableting output and production cost. Maximizing the tableting rate without compromising tablet quality is a key objective in tablet production. It should be noted that the compaction cycle will be shorter at higher tableting rates and cause a reduction in the dwell time [32]. This reduces the time given to the time-dependent plastic deformation of compacted materials, such as MCC, and may result in the formation of weaker tablets. Although a higher tableting rate has been reported to be associated with greater tablet weight variation for MUPS tablets [27], the influence of the tableting rate on MUPS tablets has not been well researched.

The compaction of sustained release coated pellets into MUPS tablets has been reported to cause compaction-induced pellet coat damage that impairs the desired sustained release function of the dosage form [4,5,6,7,10,11,12,13,33,34,35,36]. This issue has often been mitigated using formulation-based strategies. Briefly, these strategies include incorporating a cushioning agent into the filler material [7,9,13], including cushioning pellets [5,37,38], or overcoating pellets with filler material or lubricant [39,40]. However, the application of compaction parameters to mitigate pellet coat damage in MUPS tablets has not been well explored. Some studies reported favorable effects of longer dwell time in MUPS tableting [5,13]. It was also reported that a higher main compaction force, higher precompression force, and faster tableting rate adversely impacted the quality of MUPS tablets containing enteric coated pellets [27]. Higher compaction pressures led to greater pellet coat damage, but a sufficiently high compaction pressure is needed to yield a tablet of sufficient strength to endure hazards associated with post-compaction processing [41]. Hence, it is of interest to evaluate the influences of compaction parameters on both pellet coat damage and tablet tensile strength. In previous studies, it was shown that increasing the dwell time from 10 to 60 ms did not result in any significant increase in pellet coat damage [5,13]. In another study, the main compression was reported to be highly important, whereas the precompression was comparatively less important for both the formation of a strong MUPS tablet and the extent of enteric pellet coat damage [27]. The tablet strengthening effect with increased dwell time [42] and time dependent behavior [43] of tablets containing MCC have also been reported.

Multilayering is a technique to prepare tablets with two or more distinct layers that comprise different materials [44,45,46,47,48,49,50,51,52]. It has been shown that this technique is useful in the formulation of incompatible drugs into the same dosage form [53]. Various important factors have been studied for the formulation of a multilayered tablet [45,47,52]. A multilayer tableting approach could be useful in MUPS tableting, as previous studies have demonstrated that pellet-punch contacts can exert considerable pellet coat damage during MUPS tableting [11,34]. The feasibility and effectiveness of multilayering to mitigate pellet-punch effects have not been well explored and is therefore of interest.

This present study was sub-divided into three parts to directly address the research gaps of the impacts that precompression, trilayering and tableting rate have on the compaction of sustained release coated pellets into MUPS tablets. Precompression was evaluated at 0 (no precompression), 7.5 and 15.0 MPa, with the main compression pressure set at 40 MPa to yield tablets with adequate strength and extent of pellet coat damage [11,12,33]. These precompression values were selected to compare three equally spaced levels in precompression and establish potential linear relationships during analysis. The usefulness of trilayering for MUPS tablets was investigated by incorporating cushioning layers without pellets at the top and bottom of the MUPS tablet and the resultant layered tablet was compared with a normal MUPS tablet prepared at similar compaction pressures, 20 or 40 MPa. These two compaction pressure levels were selected to evaluate whether trilayering could mitigate compaction-induced pellet coat damage related to risk factors in MUPS tablets such as pellet-tooling effects that are exacerbated at higher compaction pressure [11,13]. The tableting rates of 20, 60, or 100 rpm in a compaction simulator were used to prepare MUPS tablets at different output levels and tablet target thicknesses. The MUPS tablets contained pellets coated with either ethylcellulose (EC) or acrylic polymer (AC). These pellets were composed of inert sugar cores layered with drug below an EC or AC outer coat. The key MUPS tablet properties that were evaluated include the tensile strength, disintegration time, extent of pellet coat damage, and friability depending on the compaction parameters and techniques used. An alcohol dumping study was not performed, which may be considered a limitation of the study.

## 2. Materials and Methods

### 2.1. Materials

Metformin hydrochloride (Granules India Limited, Hyderabad, India) was the model drug used. Sugar cores (Suglets^®^ 500–600 μm, Colorcon, Harleysville, PA, USA) were drug-layered before a sustained release overcoat was applied. Hydroxypropyl methylcellulose (Methocel VLV, Dow Chemical, Midland, MI, USA) and polyvinylpyrrolidone (Plasdone C-15, Delaware, DE, USA) were used to layer metformin hydrochloride onto the sugar cores. The sugar cores were selected to yield pellets with an approximate size of 700 μm. Metformin hydrochloride was used as the model drug because its high solubility in water would facilitate detection of pellet coat damage [54]. For the EC pellet coat type, an aqueous EC dispersion (Surelease^®^, Colorcon, USA) was used. For the AC pellet coat type, an aqueous poly(meth)acrylates dispersion (Eudragit RS30D, Evonik, Essen, Germany) was used with triethyl citrate (Merck, Darmstadt, Germany) as plasticizer and talc (Chemipure, Singapore) as anti-tacking agent. MCC (Ceolus PH-101, Asahi Kasei, Tokyo, Japan), silicified MCC (SMCC; Prosolv^®^ SMCC 50, JRS Pharma, Patterson, NY, USA), polyethylene glycol 3350 (Clariant, Munich, Germany), vinylpyrrolidone-vinyl acetate copolymer (Kollidon^®^ VA 64 Fine, BASF, Ludwigshafen, Germany), sodium starch glycolate (Explotab^®^, JRS Pharma, USA) and sodium stearyl fumarate (SSF; PRUV^®^, JRS Pharma, USA) were used to prepare the filler material for the tablets. SSF was also used to lubricate the EC pellets, where appropriate. Purified water was used for the disintegration tests and degassed purified water was selected as the medium for the dissolution tests following the procedure of previous studies on pellet coat damage [4,5,6,7,10,11,12,33,34,55,56].

### 2.2. Preparation of Sustained Release Coated Pellets

The preparation of sustained release coated pellets followed the methods reported in a preceding study [11]. Section 2.2.1, Section 2.2.2 and Section 2.2.3 below summarize these methods.

#### 2.2.1. Preparation of Drug-Layered Pellets

Sugar cores weighing 1 kg were first layered with metformin hydrochloride solution using a tangential air suspension coater (FlexStream^TM^ module, MP-1, GEA, Düsseldorf, Germany) to a 75%, *w*/*w* weight gain of the drug layer. Subsequently, the pellets were dried at 60 °C in a hot air oven for 12 h before sieving to obtain a 500–1000 μm size fraction. Fractionated pellets were subsequently coated with a polymer to confer sustained release function.

#### 2.2.2. Preparation of AC Pellets

Drug-layered pellets were coated with AC in a bottom spray Wurster fluid bed coater (Strea-1, GEA, Germany) to a 10%, *w*/*w* weight gain. A bottom spray coater was used for sustained release coating in lieu of a tangential air suspension coater due to it being more appropriate for the smaller batch size of 200 g compared to 1 kg. An inlet air temperature of 40 °C and a spray rate of 8 g/min were used. AC pellets were cured at 40 °C in a hot air oven for 12 h. AC pellets were then sifted to yield a 500–1000 μm size fraction for further testing.

#### 2.2.3. Preparation of EC Pellets

Drug-layered pellets were coated with EC as detailed in Section 2.2.2 for AC pellets, but with key differences in the inlet air temperature and spray rate, set to 60 °C and 8–10 g/min, respectively. The EC pellets were kept at 60 °C in a hot air oven for 12 h before sifting to yield a 500–1000 μm fraction for further testing.

#### 2.2.4. Lubrication of EC Pellets with SSF

EC pellets were lubricated with SSF through a dry powder layering method using predetermined conditions. Briefly, 1 g of pellets and 200 mg of SSF were weighed and transferred into a glass vial. The vial was then fitted onto an orbital shaker (IKA Vibrax VXR, Janke & Kunkel, Wilmington, NC, USA) and the pellet-SSF mixture was shaken at 600 rpm for 6 h to ensure proper layering with SSF. The lubricated pellets were then separated from undeposited SSF and weighed to determine the weight gain or amount of SSF deposited onto the pellets. No breakage of the coated pellets was observed in the lubrication step. No lubricated pellets were discarded, and they were randomly selected for testing. For ease of reference hereafter, lubricated EC pellets were coded as EC-1%SSF pellets, which indicates the 1% weight gain in SSF. Preliminary studies, including visual and physical inspections, were conducted on other lubricated EC pellets and qualitatively showed that SSF was successfully deposited on EC pellets through the described method.

### 2.3. Assessment of Sustained Release Coated Pellets

#### 2.3.1. Assessment of Pellet Size and Shape

Pellets size and shape were assessed using a stereomicroscope fitted to a camera (SZ61, DP71, Olympus Corporation, Tokyo, Japan) by obtaining images of 600 pellets and using imaging software (Image-Pro, Version 6.3, Media Cybernetics, USA) to measure the size and shape of pellets.

A cumulative undersize plot was generated based on the pellet size measurements and the pellet span was evaluated using Equation (1).

(1) $$Span=\frac{\left(D_{90}-D_{10}\right)}{D_{50}}$$

where D_10_, D_50_ and D_90_ represent the pellet size at the 10th, 50th, and 90th percentile, respectively. A higher span value represents a wider distribution of the pellet sizes.

The pellet aspect ratio and roundness were assessed to evaluate the pellet shape. The aspect ratio was calculated using Equation (2) and is a measure of the pellet elongation.

(2) $$Aspect\;ratio=\frac{l}{b}$$

where *l* represents the distance between the two most distant points of the two-dimensional pellet outline and *b* represents the breadth which is perpendicular against *l*.

The roundness of pellets was assessed using Equation (3), where a roundness value nearer to unity represents the pellet shape being close to a perfect circle.

(3) $$Roundness=\frac{P^{2}}{4\pi A}$$

where *P* and *A* are the perimeter and area of the pellet, respectively.

#### 2.3.2. Assessment of Pellet Crushing Strength, Elastic Modulus, and Compression Energy

The pellet crushing characteristics were evaluated through diametrical compression between two platens fitted to a universal tester (EZ-Tester-100 N, Shimadzu, Kyoto, Japan) as reported previously [11]. Pellets were sieved through a sieve with a known aperture size (Endecotts, Hope Valley, UK) to yield pellets of equal size for testing. Pellets that initially passed through were sieved for a second time using the same sieve. Pellets retained on the sieve mesh in the second sifting were collected for the pellet crushing test. The maximum load (F_m_) required to crush the pellet at a rate of 0.5 mm/min was recorded. The crushing rate was selected to characterize the crushing characteristics of a pellet with an approximate size of 700 μm. The crushing strength of a pellet was then assessed using Equation (4).

(4) $$Crushing\;strength=4\times \frac{F_{m}}{\pi d^{2}}$$

where *d* is the known sieve aperture size (710 μm) and represents the pellet diameter [57]. The elastic modulus of pellets was assessed using a pellet crushing force range of 0.5 to 3 N. The pellet compression energy, which represents the energy consumed to break the pellet, was also assessed. 20 pellets in total were assessed before the results were averaged.

#### 2.3.3. Estimations of Pellet Proportional Compositions

The proportional compositions of the pellets were estimated based on the D_50_ sizes of the sugar cores, drug-layered pellets, and sustained release coated pellets, which were obtained as described in Section 2.3.1. The thickness of the sustained release coat and drug layer were estimated using Equations (5) and (6), respectively.

(5) $$Sustained\;release\;coat\;thickness=\frac{\left(Sustained\;release\;D_{50}-Drug\;layered\;D_{50}\right)}{2}$$

(6) $$Drug\;layer\;thickness=\frac{\left(Drug\;layered\;D_{50}-Sugar\;core\;D_{50}\right)}{2}$$

The individual pellet weights were estimated by weighing 100 pellets and dividing the total weight by 100 as per Equation (7). As mentioned in Section 2.2, the pellet coating weight gain was 10%, *w*/*w* of the drug-layered sugar core and the drug layer weight gain was 75%, *w*/*w* of the blank sugar core. Subsequently, the weight of an individual pellet and the pellet coating weight gain were used to estimate the pellet sustained release coat weight as per Equation (8), weight of the sugar core as per Equation (9), and drug layer weight as per Equation (10).

(7) $$Individual\;pellet\;weight=\frac{Total\;weight\;of\;100\;pellets}{100}$$

(8) $$Pellet\;coat\;weight=Individual\;pellet\;weight-\frac{Individual\;pellet\;weight}{1.1}\;$$

(9) $$Pellet\;sugar\;core\;weight=\frac{\left(Individual\;pellet\;weight-pellet\;coat\;weight\right)}{1.75}$$

(10) Pellet drug layer weight=Individual pellet weight−pellet coat weight−pellet sugar core weight

The relative weights of the components of the pellets (i.e., sugar core, drug layer, and sustained release coat) were determined to describe the pellet composition.

### 2.4. Preparation MUPS Tablets

#### 2.4.1. Preparation of Filler Material and Pellet-Binder Blend

The filler material, which consisted of various excipients, was prepared by combining and mixing the components (excluding pellets) listed in Table 1 using a cuboid blending vessel fitted with three prongs attached to a universal drive unit (AR401, Erweka, Langen, Germany) at 15 rpm for 20 min. Pellets were manually mixed with this filler material to produce the MUPS tablets individually for assessment of precompression and trilayering, because this required small batch sizes with a small amount of material totaling a few grams. For the assessment of tableting rate, which required a larger number of tablets (i.e., greater filler-pellet mass of hundreds of grams to adequately feed the material through the gravity feeder), individual MUPS tablet fabrication was not feasible and hence the filler and pellets were mixed together in the cuboid blending vessel as described above. The same cuboid blender was used across different batches.

#### 2.4.2. Preparation of MUPS Tablets to Assess Precompression

An amount of 60 mg of pellets was weighed and combined with filler material weighing 190 mg to constitute a 250 mg MUPS tablet. This corresponds to approximately 200 pellets. All pellet-filler blends were mixed well before filling the mixture into a die fitted into a compaction simulator (Styl’One Evolution, Medelpharm, Beynost, France) and care was taken to avoid pellet-filler segregation. Compaction was then carried out at a predetermined compaction pressure and using the default single compaction cycle in the Analis software (Analis, V2.08.5, Medelpharm, France) when no precompression was used. The default compaction cycle in the Analis software consists of linear rise and fall phases of the punch each set to a duration of 80 ms. For tablets produced with precompression, a double compaction cycle was used to incorporate a precompression cycle ahead of the main compression. The punches used were round and flat faced with a diameter of 10 mm (Natoli Engineering, Saint Charles, MO, USA).

#### 2.4.3. Preparation of Trilayered MUPS Tablets

**Figure 1:**
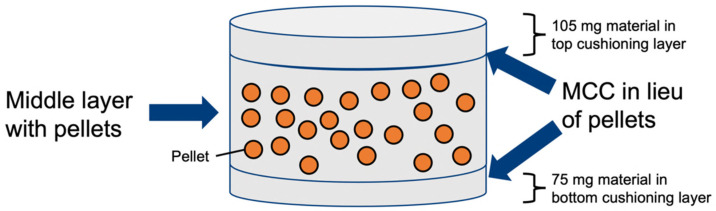
Schematic diagram of a trilayered MUPS tablet.

Trilayered MUPS tablets were fabricated using a three-stage filling process without layer tamping where a bottom cushioning layer was filled in first. The lower punch was then lowered before the pellet-filler blend was manually added as described in Section 2.4.2. The lower punch was then lowered further, and a top cushioning layer was added. The bottom and top cushioning layers weighed 75 mg and 105 mg, respectively, as depicted in Figure 1.

The die filling heights were selected depending on the volume of the formulation used and to yield tablets with consistent weights. Due to this filling technique and movement of the punches between filling stages, it was not possible to consistently produce tablets with a bottom and top cushioning layer of equal weight, hence the cushioning layers resulted in different weights (i.e., 75 mg and 105 mg). Compaction was then carried out at a predetermined compaction pressure using the same punches used to assess precompression. The components of the trilayered MUPS tablets are detailed in Table 1, where the compositions of a MCC tablet and cushioning layer were used.

#### 2.4.4. Preparation of MUPS Tablets to Assess Tableting Rate

The pellet-filler blend was added to a gravity feeder fitted in the compaction simulator, with the formulation used resembling that of the MCC tablets (Table 1). The concentration of SSF as tablet lubricant was selected at the lowest reported level of 0.5% [58]. The compaction cycle used to assess tableting rate was a simulation of a rotary tablet press (R190FT, GEA-Courtoy, Halle, Belgium), which was selected due to its previous use in another study on MUPS tablets [13]. The punches used were round, with a flat faced radius edge configuration, and of 10 mm diameter (Natoli Engineering, USA). The precompression and main compression cycles were both set using the target compression thickness setting where lower values represent a reduced compact thickness or greater level of compact compression. For the compaction simulator used in this study, only the target tablet thickness can be adjusted in lieu of compaction force when simulating a rotary tablet press. The three levels of tableting rate, measured in rpm, that were evaluated and their corresponding levels of dwell time and tableting output are listed in Table 2.

### 2.5. Evaluation of MUPS Tablets

#### 2.5.1. Assessment of Pellet Volume Fraction

The MUPS tablet apparent volume (*V_MUPS_*) was calculated using Equation (11).

(11) $$\;V_{MUPS}=\pi r^{2}h$$

where *r* and *h* represent the tablet radius and tablet thickness, respectively.

The pellet volume fraction measures the total volume of pellets relative to the MUPS tablet volume (*V_MUPS_*). The pellet volume fraction was determined using Equations (12) and (13) based on a method described in preceding studies [4,12,13].

(12) $$Pellet\;volume\;fraction=\frac{V_{p}}{V_{MUPS}}\times 100$$

(13) $$V_{p}=\frac{pellet\;mass}{p_{t}}\;$$

where *p_t_* represents the true density of pellets, which was measured in triplicate in a helium displacement pycnometer (Penta-Pycnometer, Anton Paar, Graz, Austria). *V_p_* represents the true volume of uncompacted pellets and was determined using Equation (13). At least five replicates were performed and the subsequent results were averaged.

#### 2.5.2. Assessment of Tablet Mechanical Strength

Tablet breaking force (F_T_) was determined using a tablet hardness tester (TBF 1000, Copley Scientific, Nottingham, UK). The tablet tensile strength was determined as described in a preceding report [59] using Equation (14). Five replicates were performed and results averaged.

(14) $$Tensile\;strength=\frac{2F_{T}}{\pi hD_{tablet}}$$

where D_tablet_ represents the tablet diameter.

#### 2.5.3. Assessment of Tablet Disintegration Time 

Tablet disintegration tests were performed in a disintegration tester (PTZ Auto 2, PharmaTest, Hainburg, Germany) according to the United States Pharmacopoeia (USP) method in purified water maintained at 37 °C without the use of disks. Five replicates were performed and results were averaged. The disintegration time is indicative of when the MUPS tablet starts to behave as a multi-unit preparation [14]. 

#### 2.5.4. Assessment of Drug Release

The dissolution test of the MUPS tablets or uncompacted pellets was conducted using a USP Apparatus 2 (VK7010, Varian, Palo Alto, CA, USA). The paddle was rotated at 100 rpm and the medium was 500 mL degassed purified water maintained at 37 °C. A filtered aliquot of 5 mL was withdrawn without replacement at predetermined time points of 0, 15, 30, 60, 90, 120, 180, and 240 min. After taking the sample of the last time point of 240 min, paddle rotation was paused and the pellets were completely crushed using a glass rod. Agitation was then performed for an additional 15 min with the paddles rotated at 220 rpm before a final sample was collected. The amount of drug in each sample was determined using a spectrophotometer (UV-5100, Hitachi, Tokyo, Japan) using a wavelength of 232 nm and drug concentrations corrected for the sample volume removed. At least four replicates were performed, and the results were averaged to establish the dissolution profiles.

The mean dissolution time (MDT) was calculated using Equation (15) as it represents the drug release rate and dosage form sustained release function [60].

(15) $$MDT=\frac{\sum_{i=1}^{n}{\bar{t}}_{i}\;{\times ∆M}_{i}\;}{\sum_{i=1}^{n}\;{∆M}_{i}}$$

where ${\bar{t}}_{i}$ is the midpoint of the time period during which a fraction (${∆M}_{i}$) of the drug has been released, i.e., ${\bar{t}}_{i}=\;\frac{t_{i}+t_{i+1}}{2}$ and ${∆M}_{i}=(M_{i+1}-M_{i}$). A lower MDT of compacted versus uncompacted pellets indicates a faster drug release and thus, a higher extent of pellet coat damage. The MDT of uncompacted AC and EC pellets may be different [10,11]. Hence, the MDT values of compacted MUPS tablets were converted to E_MDT_ (extent of pellet coat damage indicated by a reduction in MDT) values using Equation (16) to facilitate meaningful comparisons between the different pellets. A higher E_MDT_ value represents a larger extent of pellet coat damage.

(16) $$E_{MDT}=\frac{\left({MDT}_{UC}-{MDT}_{C}\right)}{{MDT}_{UC}}\times 100$$

where MDT_C_ and MDT_UC_ represent the MDT of compacted and uncompacted pellets, respectively. The focus of analyses of pellet coat damage in the present study centers around the E_MDT_ values, hence the dissolution profiles are included in Appendix A–Appendix C, for reference.

The impact of lubricating EC pellets with SSF on the dissolution of uncompacted pellets was assessed by calculating the similarity factor (*f*2) using Equation (17), which compares the similarity of two dissolution profiles using the relative error at each time point.

(17) $$f2=50\times \log\left\{{\left[1+\left(\frac{1}{n}\right)\;\sum_{i=1}^{n}{\;\left|R_{i}-T_{i}\right|}^{2}\right]}^{-0.5}\times 100\right\}$$

where *n* represents the number of time intervals in which samples were withdrawn, *R_i_* is the mean percentage of drug release for the uncompacted EC pellets at time interval *i* and *T_i_* is the percentage drug release for a single replicate of uncompacted EC pellets lubricated with SSF at time interval *i*. For calculations of the *f*2 for uncompacted pellets, at least 6 replicates of each were carried out. An *f*2 value above 50 indicates similarity of the two dissolution profiles.

### 2.6. Assessment of Compaction Energy Parameters

The compaction energy values were derived from a force displacement curve generated within the compaction software (Analis, V2.08.5, Medelpharm, France). As described in preceding reports, various compaction energy parameters can be derived from this force displacement curve [12,61,62,63]. In the present study, the rearrangement and plastic energy values were evaluated. The rearrangement energy refers to the energy consumed when particles repack tightly in the die without excessive deformation [64,65]. The plastic energy refers to the energy taken up to deform the compacted material irreversibly when particle rearrangement has ceased.

### 2.7. Statistical Analysis

One-way analysis of variance (ANOVA) was used to determine significant difference between groups by comparing their specified means, while differences between two means were compared through an independent t-test. Linear regression was performed on the tableting rate data. A significance level of *p* = 0.05 was employed. The statistical analysis was conducted with the aid of a statistical software (Minitab, Version 18, State College, PA, USA).

## 3. Results and Discussion

### 3.1. Characterization of Coated Pellets

The characteristics of the EC and AC pellets were determined (Table 3). The EC and AC pellets had comparable aspect ratio, roundness, D_50_, and span values. The AC pellets had higher true density than the EC pellets. The thicknesses of the various layers contained within the pellet (i.e., pellet coat and drug layer) and the relative weights of the components were estimated. Notably, the EC pellet coat was thicker than the AC pellet coat. Moreover, the AC pellets had slightly higher crushing strength than the EC pellets, but this difference in crushing strength was not statistically significant (*p* = 0.243). However, the elastic modulus value of the AC pellets was significantly higher (*p* = 0.007), which indicates that the AC pellet coat was comparatively more resistant to deformation and mechanically stronger than the EC pellet coat. The pellet compression energy values were not significantly different (*p* = 0.127) between the EC and AC pellets.

In a previous study [66], EC films were reported to be hard and brittle in the dry state but soft when wetted. On the other hand, AC films were reported to be flexible in both the dry and wet states. The AC films also possessed a higher puncture strength than the EC films [66]. The pellet property results (Table 3) collectively indicate that the pellet crushing strength and compression energy are dependent on the nature of the pellet core, which in this study was composed of a drug-layered sugar core that forms the majority of the pellet by weight (Table 3). On the other hand, the elastic modulus is dependent on the mechanical properties of the sustained release coat, which may be influenced by plasticizer in the pellet coats. For AC pellets, triethyl citrate comprised roughly 5%, *w*/*w* of the AC pellet coat and 0.5%, *w*/*w* of the total AC pellet, whereas in EC pellets plasticizers such as oleic acid and medium chain triglycerides are already included in the commercially available Surelease dispersion [67,68] and no additional plasticizer was added in the EC coat in the present study. The AC and EC pellets were tested and used under the same ambient temperature and relative humidity conditions.

Overall, due to the similar morphology and characteristics of EC and AC pellets, any resulting differences in the extent of pellet coat damage measured may be attributable to the different mechanical nature of the EC and AC pellet coats, as indicated by the difference in elastic modulus and the differences in mechanisms through which each pellet coat type experiences damage as a pellet coat [11]. A previous study of pellets compacted into MUPS tablets similar to those used in the present study showed that EC pellet coats were more susceptible to cracking rather than deformation, whereas the opposite applied to AC pellet coats [11].

### 3.2. Impact of Precompression on MUPS Tablets

The pellet volume fraction has been shown to affect the extent of pellet coat damage in MUPS tablets [4,5,12,13] and the influence of including precompression on the pellet volume fraction values was therefore evaluated (Table 4).

The pellet volume fraction values of all MUPS tablets in the present study were below 18% and thus, well below the critical threshold of 30% [4,12,13]. Consequently, this would limit the extent of pellet coat damage due to previously studied risk factors in MUPS tableting [11], and would enable the elucidation of the effects that precompression and the other compaction parameters could have on pellet coat damage. The pellet volume fraction values were not significantly (ANOVA, *p* > 0.05) different with the same filler material, which shows that the precompression did not appreciably affect the pellet volume fraction values. Precompression did not reduce the overall volume of the MUPS tablets (shown in Table A1 in Appendix A) significantly, which aptly accounted for the above observation. Therefore, any difference in pellet coat damage with a different level of precompression could be related to the change in pellet-filler interface rather than volume-related effects.

**Figure 2:**
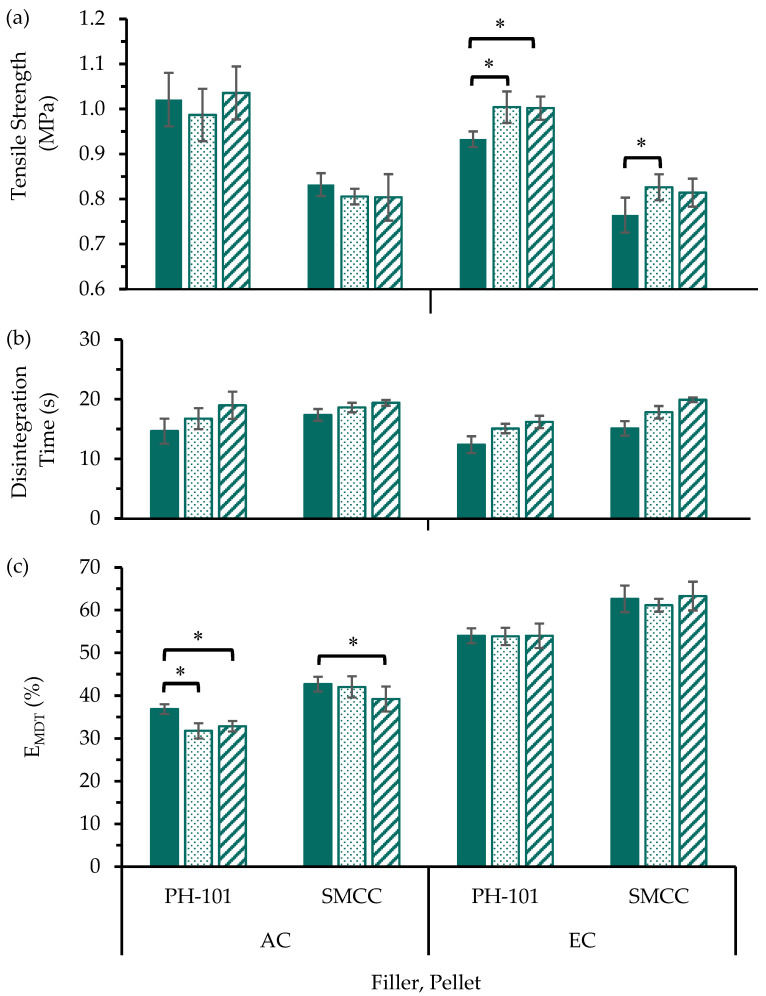
(**a**) Tensile strength, (**b**) disintegration time, and (**c**) E_MDT_ values of MUPS tablets prepared without precompression (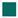) or with precompression of either 7.5 (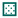) or 15.0 MPa (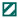) before compaction at 40 MPa. Significant differences are denoted by * and some are omitted for visual clarity.

The influences of precompression on the MUPS tablet tensile strength were evaluated (Figure 2a) with dissolution profiles charted in Appendix A. For MUPS tablets containing AC pellets, there appeared to be no influence of precompression on the MUPS tablet tensile strength. On the contrary, the tensile strength of the MUPS tablets containing EC pellets was higher with the inclusion of precompression. However, increasing the precompression level from 7.5 to 15.0 MPa when using PH-101 in the filler material did not result in significantly (*p* > 0.05) higher tensile strength. Therefore, these results suggest that a precompression of 7.5 MPa would be sufficient to increase the MUPS tablet tensile strength.

**Figure 3:**
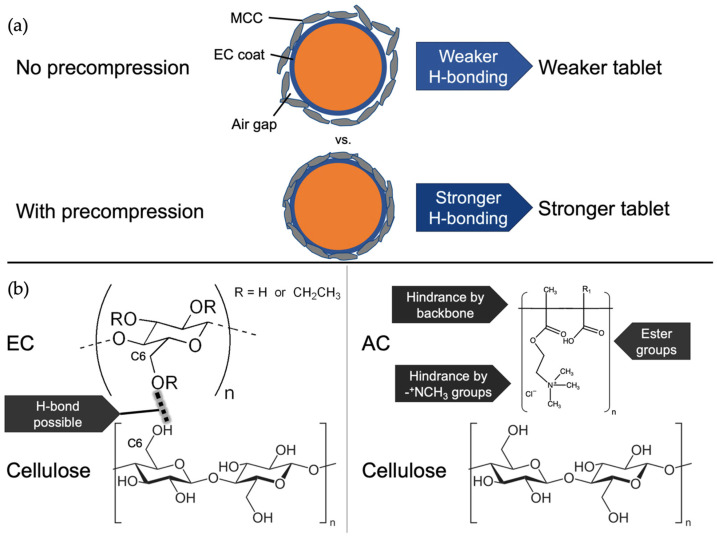
Schematic diagrams illustrating the (**a**) MCC-EC interactions without or with precompression and (**b**) molecular structures and potential mechanisms of hydrogen bonding of EC and AC with MCC.

The contrasting influences of precompression on MUPS tablets containing AC or EC pellets suggest that the tablet strengthening effect of precompression is related to or influenced by the interactions between the pellet coat and the filler material. Potentially, hydrogen bonding between the EC polymer on the pellet coat and the filler MCC particles [69,70,71,72] could be enhanced in MUPS tablets produced with precompression due to a removal of air [24] between the MCC particles and pellets before compaction, which could result in the formation of a stronger MUPS tablet after compaction as depicted in Figure 3a. As shown in Figure 3b, the feasibility of hydrogen bonding would be greater with EC polymer due to available oxygen moieties compared to AC due to potential hindrance by the polymer backbone and quaternary ammonium groups of the latter polymer type. Collectively with the similar pellet dimensions (Table 3) and proportions per tablet (Table 4), this postulation could explain why the tablet strengthening effect of precompression was only observed for the EC pellets. Although different types of bonding such as van der Waals, covalent bonds, ionic bonds and mechanical interlocking are also possible between EC and AC pellet coats with MCC, the available literature [69,70,71,72] points toward the possibility of hydrogen bonding being responsible for the observed differences, as the other bond types would be much weaker than hydrogen bonding [73]. This influence of hydrogen bonding between EC and MCC was further investigated and discussed in Section 3.3.

The tablet tensile strength values of MUPS tablets containing PH-101 were significantly (*p* < 0.05) higher by 0.17–0.23 MPa than those containing SMCC in the filler material (Figure 2a). This difference could be attributed to the silicification of the MCC particles by the colloidal silicon dioxide in SMCC, thereby reducing the binding capability of the bulk MCC particles [58,74]. Based on these MUPS tablet tensile strength results, PH-101 would be a better filler than SMCC for MUPS tablets.

The disintegration times of the MUPS tablets were also determined (Figure 2b) and show that all MUPS tablets disintegrated within 30 s. This rapid tablet disintegration can be attributed to the disintegrant effect of sodium starch glycolate [34,75,76]. However, there were weak trends showing that including and increasing the level of precompression led to increases in tablet disintegration time, but these differences were small relative to the 4 h dissolution run. These differences notwithstanding, the tablet disintegration should not confound the drug release used to determine the extent of pellet coat damage.

The E_MDT_ values (Figure 2c) were determined to evaluate the influence of precompression on the extent of pellet coat damage based on the dissolution profiles (Appendix A). The results for the EC pellets show that the inclusion and increase in precompression exerted a negligible effect on the damage to the EC pellets. However, for AC pellets, the E_MDT_ values were significantly lower (*p* < 0.05) when precompression was included at both 7.5 and 15.0 MPa compared to when no precompression was used with PH-101 in the filler material. For MUPS tablets with AC pellets and SMCC in the filler material, the E_MDT_ values at the higher precompression level of 15.0 MPa were significantly (ANOVA, *p* < 0.05) lower than when no precompression was used. This indicates that for AC pellets, precompression appeared to exert a small protective effect on the AC pellet coats and that this protective effect was greater when PH-101 was in the filler material. The main difference between the PH-101 and SMCC filler materials is the silicification of the MCC particles in the SMCC material [58,74]. This silicification has been associated with glidant properties which can improve the flow of the bulk material [58,77,78]. An earlier study reported that the ‘silification’ of MCC produced a material (i.e., SMCC) that was chemically and physically ‘very similar to standard MCC’ [74]. However, in another study [79], the mechanical properties of compacts comprising SMCC and MCC were found to be different, with SMCC compacts being stronger. Nevertheless, in the present study, MUPS tablets consisting of SMCC were found to have lower tensile strength than the corresponding MUPS tablets consisting of PH-101, suggesting significant influence of the pellets on the tablet properties. Overall, the findings suggest that the addition of colloidal silicon dioxide could have reduced the interparticulate friction that has been linked to reduced pellet coat damage during compaction [10,11,12], but this could also cause the resultant transfer of compaction stress to the pellet coats to be increased with SMCC and ultimately cause greater pellet coat damage compared to when PH-101 is used.

The lower E_MDT_ values (Figure 2c) and higher MUPS tablet tensile strength values (Figure 2a) when PH-101 was used as the filler material compared to when SMCC was used demonstrate that PH-101 was superior compared to SMCC as a filler material for MUPS tablets. Therefore, it can be concluded from the data in the present study that PH-101 is a better filler material for MUPS tableting for both AC and EC pellets. As such, SMCC was not used in the further studies detailed in subsequent sections herein.

The E_MDT_ values (Figure 2c) also demonstrate that the EC pellets exhibited significantly (ANOVA, *p* < 0.05) more pellet coat damage than the AC pellets with both PH-101 and SMCC as the filler material and at all precompression levels. This can be attributed to the stronger mechanical strength of the AC pellet coats as indicated by the significantly higher elastic modulus of the AC pellets (Table 3) and more brittle nature of EC polymer as a film [80,81]. These results further demonstrate that AC is a comparatively more suitable polymer for MUPS tableting due to the higher resistance against compaction-induced pellet coat damage.

**Figure 4:**
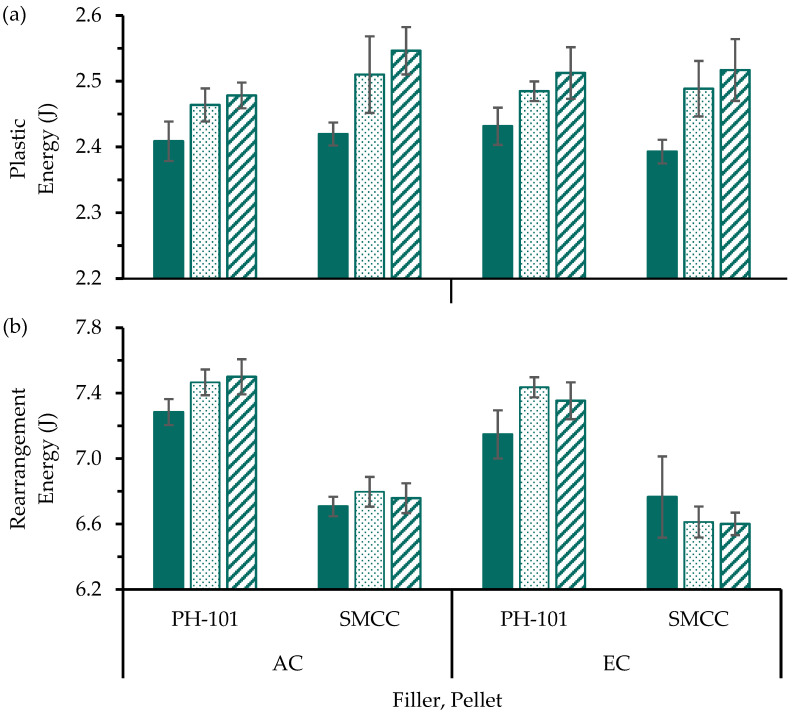
(**a**) Plastic and (**b**) rearrangement energy values of MUPS tablets prepared without precompression (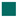) or with a precompression of either 7.5 (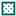) or 15.0 MPa (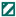) before compaction at 40 MPa. Significant differences are omitted for visual clarity.

The plastic and rearrangement energy values (Figure 4) were determined to explore the mechanisms that could influence the differences in tablet tensile strength and pellet coat damage. The most notable differences were the lower rearrangement energy values of the tablets fabricated with SMCC compared to PH-101 in the filler material. These results further substantiated that the silicification of MCC particles could lead to a reduction in the interparticle friction due to the glidant properties of colloidal silicon dioxide. Consequently, the total energy sequestered by the filler particles rearranging is comparatively less for SMCC than PH-101, supporting the aforementioned notion that increased stress transfer to the pellet coats caused the observed increase in pellet coat damage [10].

### 3.3. Impact of Trilayering on MUPS Tablets

**Figure 5:**
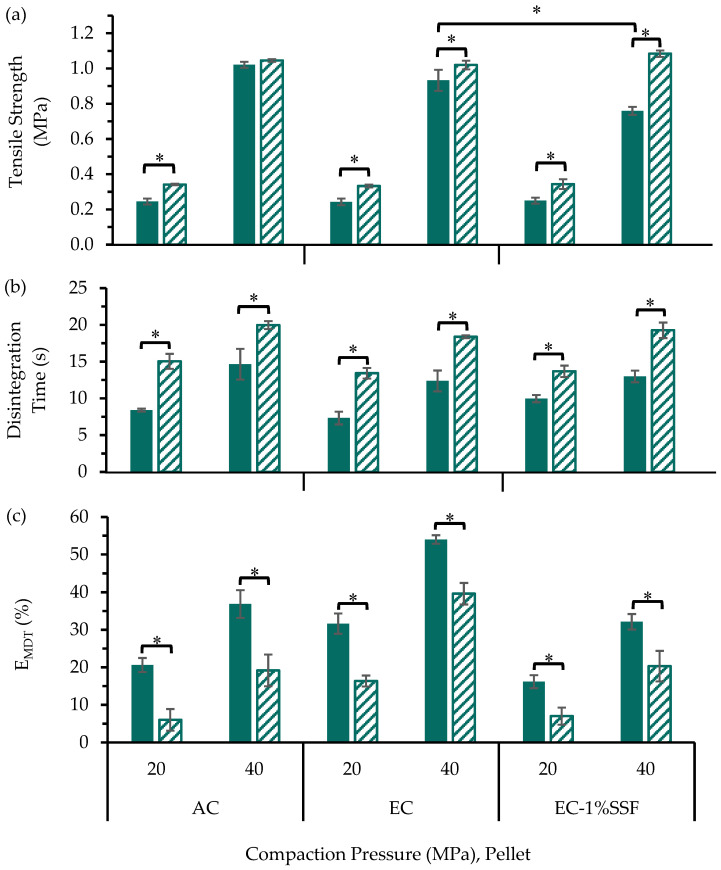
(**a**) Tensile strength, (**b**) disintegration time, and (**c**) EMDT values of normal (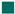) and trilayered (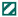) MUPS tablets. Significant differences are denoted by * and some are omitted for visual clarity. PH-101 was used in the filler material.

The tensile strength of normal and trilayered MUPS may differ due to the inclusion of cushioning layers. These cushioning layers are devoid of pellets. When compacted, they may be stronger than the regions of the tablet that contain pellets [12]. The tablet tensile strength values (Figure 5a) show that the trilayered MUPS tablets were significantly (ANOVA, *p* < 0.05) stronger than the normal MUPS tablets, except when AC pellets were compacted at 40 MPa. Therefore, it can be concluded that the cushioning layers generally strengthened the overall MUPS tablet due to their lack of pellets. The underlying mechanism could be that the filler particles in the cushioning layers exhibit stronger interparticle bonds and greater plastic deformation [12]. The strengthening effect of trilayering would enable the use of a lower compaction pressure to produce the MUPS tablets, which could further reduce the extent of compaction-induced pellet coat damage [10,11,12].

The tablet disintegration values (Figure 5b) show that all tablets disintegrated within 25 s. Pellet shielding by the cushioning layers should therefore not confound the drug release used to assess the extent of pellet coat damage. However, it was evident that the disintegration times of the trilayered MUPS tablets (430 mg) was longer than those of the normal MUPS tablets (250 mg). This result can be attributed to the larger size of the trilayered MUPS tablets and their greater mass, which requires a longer time for complete disintegration to occur. Although trilayered MUPS tablets took longer to disintegrate, this difference is minimal with respect to the dissolution time of 4 h. Tablet disintegration is therefore not a considerable disadvantage of trilayering MUPS tablets. This can be explained by the use of sodium starch glycolate at a concentration of 1%, *w*/*w*, facilitating rapid tablet disintegration [34,75,76] in all MUPS tablet layers.

**Figure 6:**
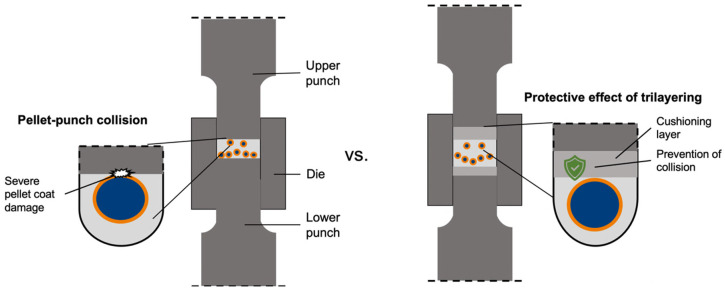
Schematic diagram depicting the theoretical protective effect of trilayering a MUPS tablet. Left: normal MUPS tablet compaction. Right: trilayered MUPS tablet compaction. 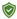: protection against direct contact between the pellet and punch face surface.

The E_MDT_ values of normal and trilayered MUPS tablets (Figure 5c) were determined to evaluate whether the cushioning layers could mitigate pellet coat damage caused by pellet-punch effects. The results show that the trilayered MUPS tablets exhibited significantly (*p* < 0.05) less pellet coat damage of 9.2–17.7% in E_MDT_ compared to the normal MUPS tablets. Therefore, the results demonstrate that the trilayering technique was able to mitigate pellet coat damage related to the pellet-punch effects as depicted in Figure 6, which would be a reduction of approximately 9.2–17.7% in E_MDT_. Moreover, the great differences of 9.2–17.7% in E_MDT_ demonstrate that the pellet-punch effects would account for a considerable proportion of pellet coat damage, up to 17.7% in E_MDT_, which supports previous postulations that the majority of pellet coat damage in MUPS tablets can be attributed to the risk factors such as pellet-punch effects, pellet-pellet effects, and pellet spatial effects rather than compaction of a pellet in filler material by itself [11,34]. The remaining amount of pellet coat damage would be attributable to pellet-filler, pellet-pellet and pellet spatial effects not mitigated by the cushioning layers.

The E_MDT_ values (Figure 5c) also demonstrate that the AC pellets exhibited significantly (*p* < 0.05) less pellet coat damage than the EC pellets regardless of tablet type due to their higher resistance to pellet coat damage. These differences agree with the previous results in this study and those in other studies where the same pellets were used but had a thinner polymer pellet coat weight gain of 8% [10,11]. It also appears that the benefit of trilayering was greater when the AC pellets were used compared to the EC pellets. For comparison, EC pellets were lubricated with SSF (EC-1% SSF) and compacted into normal or trilayered MUPS tablets. Their E_MDT_ values (Figure 5c) show that lubricating EC pellets with SSF led to significantly (*p* < 0.05) less pellet coat damage of 9.3–21.9% in E_MDT_ values. Although the MDT values of uncompacted EC and EC-1% SSF pellets were 124.51 ± 0.72 min and 126.52 ± 0.40 min, respectively, and were significantly different (*p* < 0.000), the *f*2 value comparing the two pellet types was well above 50 with a mean of 86.59 ± 2.66 and the dissolution profiles of both pellets were very similar (Appendix B). Hence, it can be presumed that EC pellet lubrication with SSF did not impede drug dissolution to a meaningful extent as to confound the E_MDT_ results after compaction. The results suggest that the potential hydrogen bonding between MCC-EC [69,70,71,72] could have been reduced by the SSF layer, thus reducing the stress transferred from the MCC particles during compaction and resulting in less EC pellet coat damage. The observations in the present study are consistent with a previous study in which SSF was sprayed onto EC pellets before compaction into MUPS tablets [40]. However, in the present study, only SSF was evaluated and it could be of interest to investigate other lubricants at different lubrication levels because the lubricant type and concentration were shown to influence lubricant efficiency [82]. In the present study, the EC pellets were coated with SSF by dry mixing the pellets with the lubricant. This is a simpler method but it is potentially not as effective compared to overcoating the EC pellets with SSF by spraying.

### 3.4. Impact of Tableting Rate on MUPS Tablets

**Figure 7:**
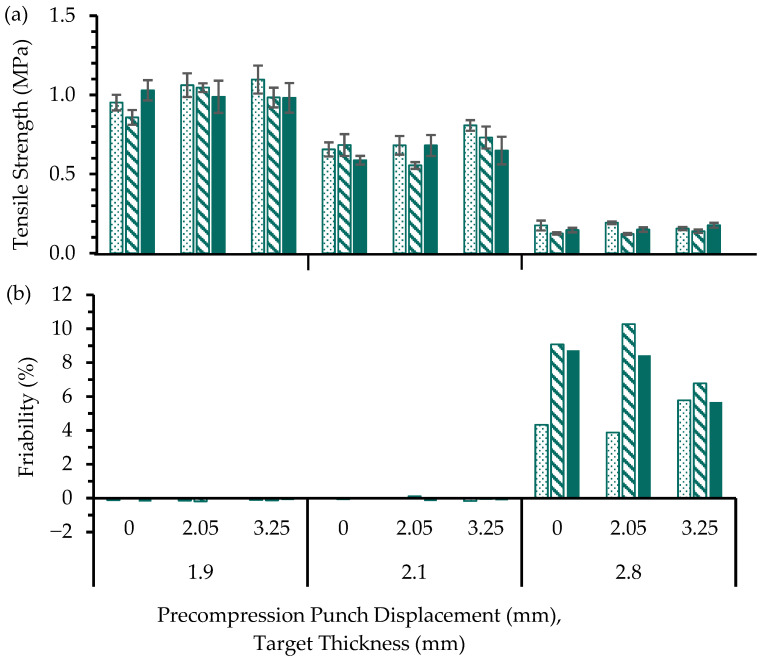
(**a**) Tensile strength and (**b**) friability values of MUPS tablets compacted with AC pellets using a tableting rate of 20 (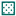), 60 (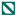), or 100 (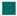) rpm. Significant differences are omitted for visual clarity. PH-101 was used in the filler material.

The tablet tensile strength values (Figure 7a) of MUPS tablets produced by the compaction simulator configured to the Courtoy R190FT rotary tablet press profile and operated at different tableting rates (Table 2) were determined. In this part of the study, only AC pellets and PH-101 were used to generate a larger dataset to assess the impact of tableting rate and precompression at various tablet target thicknesses. AC pellets were used because they were less susceptible to pellet coat damage than the EC pellets and therefore more suitable for use in MUPS tablets. As it was also of interest to determine if the tableting rate influences EC pellets, a smaller dataset for EC pellets was also obtained for comparison, where appropriate. The target thickness was set to simulate the setting of the cam shaft in the rotary tablet press, where thinner target thickness would result in greater compression of the compact and could potentially result in the formation of a stronger tablet. The results (Figure 7a) clearly show that a smaller target thickness generally yielded much stronger tablets. There is no clear trend that indicates precompression helped increase the tensile strength of the MUPS tablets at all target thicknesses. Moreover, there is no clear trend that increasing the tableting rate affected the tensile strength of the resultant MUPS tablets.

The friability values of the tablets were also determined (Figure 7b). It was apparent that the friability of the MUPS tablets produced using a target thickness of 1.9 mm and 2.1 mm had friability values near 0.0%. Therefore, these tablets passed the USP friability test which has a friability pass limit of 1.0% [83]. These friability results could be attributed to various reasons that include the use of a flat-faced radius edge punch and PH-101 in the filler material, enabling high compactibility [11,84,85] and the formation of mechanically strong MUPS tablets. However, for the MUPS tablets produced using a target thickness of 2.8 mm, the weaker tablets failed the friability test, with friability values ranging from 3.9% to 10.3%. The target thickness of 2.8 mm was therefore shown to be unable to generate sufficient compression force to produce MUPS tablets of adequate integrity.

**Figure 8:**
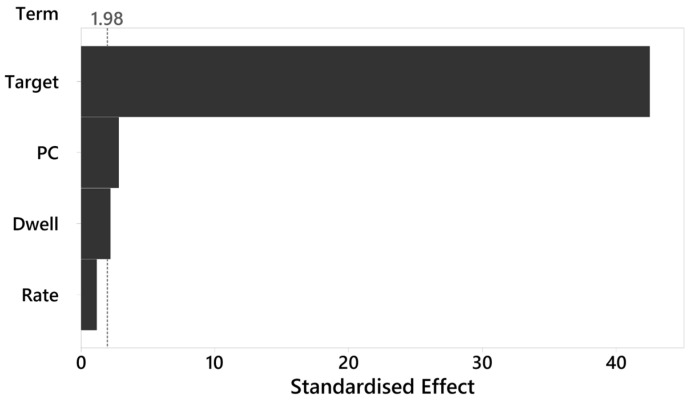
Pareto chart of standardized effects on MUPS tablet tensile strength prepared with AC pellets. PC: punch displacement for precompression; Target: target thickness; Dwell: dwell time; Rate: tableting rate. The vertical dashed line represents the significance threshold.

The influences of the precompression level, tableting rate, dwell time, and target thickness were further evaluated using a linear regression analysis (R^2^ value of 93.55%) and Pareto chart (Figure 8) depicting the standardized effect levels of these variables and illustrating the relative effects of the variables viewed together. 

The target thickness was found to be the most important and significant (*p* < 0.000) variable with the largest standardized effect and a coefficient of −0.888. This indicates that decreasing the target thickness would lead to significantly stronger MUPS tablet tensile strength, which would be the result of an increased amount of compression and plastic deformation of the compacted material. Precompression was the second most important and significant (*p* = 0.005) variable and its coefficient value of 0.017 shows that increasing the precompression level increased MUPS tablet tensile strength. It should be recalled that an earlier independent t-test showed that precompression had no obvious effect on the tensile strength of tablets with AC pellets (Figure 2a). However, it can be seen from the linear regression analysis that precompression was associated with statistically significant increases in the tensile strength of MUPS tablets containing AC pellets. The dwell time was the third significant (*p* = 0.029) variable and its coefficient of 0.005 shows that increasing the dwell time, which can be achieved through reducing the tableting rate (Table 2) or introducing a dwell time extension mechanism into the rotary tablet press [5,13], led to increased MUPS tablet tensile strength. The findings in the present study are in agreement with a previous study where decreasing the compaction speed was associated with longer dwell times, which resulted in greater tablet strength [86]. Interestingly, the tableting rate was not a significant factor (*p* = 0.235). This indicates that the formation of stronger tablets was linearly dependent on the dwell time. Nevertheless, it was observed that the dwell time was non-linearly dependent on the tableting rate (Table 2) and it can still be inferred that higher tableting rates resulted in weaker MUPS tablets due to the associated reduction in dwell time. Additionally, this also suggests that prolonging the dwell time at higher tableting rate could be beneficial in strengthening MUPS tablets.

Although precompression and dwell time were significantly associated with MUPS tablet tensile strength in the linear regression analysis, their standardized effects were small compared to the much larger effect of the target thickness variable. Therefore, if the goal is to increase the MUPS tablet tensile strength, it may be more effective to change the target thickness setting in lieu of adding a precompression cycle or increasing the amount of precompression or in conjunction reducing the tableting rate. Although decreasing the target thickness level to make a thinner compact can increase MUPS tablet tensile strength, it must be noted that the increase in compaction force caused higher pressure to be exerted on the compact as well, which in turn could also increase pellet coat damage.

**Figure 9:**
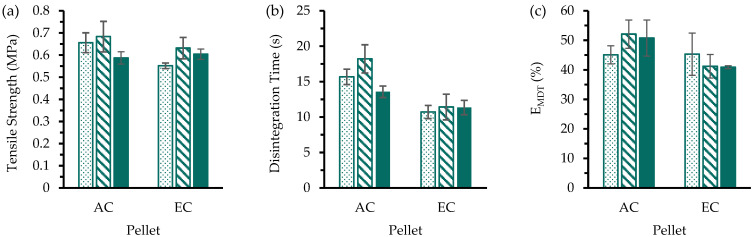
(**a**) Tensile strength, (**b**) disintegration time, and (**c**) E_MDT_ values of MUPS tablets compacted with AC or EC pellets using a tableting rate of 20 (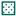), 60 (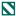), or 100 (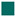) rpm to a target thickness of 2.1 mm without precompression. Significant differences are omitted for visual clarity. PH-101 was used in the filler material.

From the tensile strength and friability results (Figure 7), the extent of pellet coat damage at different tableting rates was derived for the target thickness of 2.1 mm. This target thickness was selected because tablets produced passed the friability test but this thinner target thickness could result in excessive pellet coat damage. The properties of the MUPS tablets containing AC or EC pellets produced without precompression at various tableting rates are shown in Figure 9.

The tensile strength results (Figure 9a) do not show any clear trend across both pellet types. For AC pellets, the MUPS tablet tensile strength was the lowest at the highest tableting rate of 100 rpm. In contrast, the tablet tensile strength for the EC pellets was the lowest at the lowest tableting rate of 20 rpm. These results suggest that the polymer coat could interact with the tableting rate, whereby bonding between the pellet coat and the filler particles may be affected by the nature of the bonds and the rates at which the tablets are produced. Although this effect could not be ascertained directly, previous studies demonstrated differences in tensile strength of tablets compacted with EC and AC pellets, which supports the postulation that MCC filler particles interact differently with EC and AC pellet coats upon compaction [10,11,33]. Nonetheless, the inferences of the AC pellet results may not directly be applicable to those of the EC pellets.

The disintegration times (Figure 9b) were also determined to assess whether pellet shielding could confound the drug release used to determine the extent of pellet coat damage. As all tablets disintegrated well within 25 s, tablet disintegration was likely rapid enough to avoid pellet shielding in a 4 h dissolution run and the drug release used to assess the extent of pellet coat damage should therefore not be confounded.

The E_MDT_ results (Figure 9c) based on the dissolution profiles (Appendix C) show that the tableting rate exerted minimal influence on the extent of pellet coat damage. Moreover, Equation (17) was used to determine the *f*2 values between averaged dissolution profiles for each tableting rate (i.e., 20–60, 20–100, and 60–100 rpm). Comparing all dissolution profiles per pellet, the *f*2 values ranged from 63 to 89 and thus demonstrate quantitatively that the dissolution profiles were similar to each other regardless of the tableting rate used. Overall, these results agree with a previous study where increasing the dwell time, which is shortened at higher tableting rates (Table 2), did not appreciably affect pellet coat damage [5,13]. Therefore, it is possible that the induction of pellet coat damage may not be time-dependent, even though the plastic deformation of the MCC filler material is time-dependent. Thus, from the available findings, the extent of pellet coat damage appears to be associated mainly with the magnitude of the compaction force applied.

### 3.5. Implications on the Industrialization of MUPS Tablets

The results in this study provide key insights on how compaction parameters could impact the production of MUPS tablets containing sustained release coated pellets, despite the limitation that an alcohol-dumping study was not performed. In the sub-study where MUPS tablets were produced using the default compaction cycle, precompression was found only to increase the tensile strength of MUPS tablets containing EC pellets (Figure 2a) but not of those containing AC pellets. However, in a larger dataset of the sub-study where a simulated rotary tablet press is used (Figure 8), precompression was significantly associated with increased tensile strength of MUPS tablets containing AC pellets, although the magnitude of this benefit was relatively small compared to reducing the target thickness (Figure 8). Therefore, the present results provide conflicting findings that precompression could yield benefits for MUPS tablets.

Trilayering MUPS tablets was found to be a highly effective method to both strengthen MUPS tablets (Figure 5a) and reduce pellet coat damage (Figure 5c). However, a major disadvantage of this approach for industrial MUPS tableting is the need for a customized rotary tablet press with three sequential feeding stages for the two cushioning layers and the pellet-filler mixture. This approach could increase the complexity and costs of producing trilayered MUPS tablets compared to normal MUPS tablets. Nevertheless, the results in this study demonstrate the feasibility of using the trilayering approach to mitigate pellet coat damage in MUPS tablets.

Lubricating EC pellets with SSF was associated with markedly reduced pellet coat damage (Figure 5c). It was postulated that this could be attributed to the reduction in mechanical stress transfer from the compaction force to the EC pellet coats by interrupting the hydrogen bonding between MCC filler particles and the EC. However, it is uncertain whether this technique could be employed effectively at an industrial scale.

Increasing the tableting rate is a highly valued option to improve productivity in the manufacture of MUPS tablets. However, increasing the tableting rate may lead to the formation of significantly weaker MUPS tablets caused by a shortening of the dwell time (Figure 8). Nevertheless, the extent of pellet coat damage was not appreciably increased by using a higher tableting rate (Figure 9c). Therefore, care must be taken to ensure that MUPS tablets produced at a high tableting rate possess sufficient tensile strength by adjusting the target thickness accordingly and mitigating any increased pellet coat damage arising from the correspondingly increased compaction pressure.

## 4. Conclusions

The findings of this study demonstrate that compaction parameters and techniques can impact sustained release MUPS tablet properties in a variety of ways. For example, precompression significantly strengthened MUPS tablets containing EC pellets while only reducing, albeit to a minor extent, the coat damage of AC pellets. Statistically, the use of precompression, when evaluated in a larger dataset at different tableting rates and simulating a rotary tablet press, was shown to produce stronger MUPS tablets, albeit to a much lesser extent compared to reducing the target thickness. The introduction of a cushioning layer at the bottom and at the top of the MUPS tablet, to constitute a trilayered MUPS tablet, improved the mechanical strength of the tablet and also led to considerably mitigated pellet coat damage. This protective effect was attributed to the prevention of surface pellets being damaged by interactions with the punch faces. EC pellet lubrication with SSF was shown to significantly mitigate pellet coat damage. Lastly, increasing the tableting rate led to reduced MUPS tablet tensile strength due to reduced dwell time, but did not appear to increase the extent of pellet coat damage. The findings generated in this study provide key information on the compaction of MUPS tablets that could better inform future developments of MUPS tablets.

## Figures and Tables

**Table 1:** Formulations of the tablets and cushioning layer.

Component	MCC Tablets (%, *w*/*w*)	SMCC Tablets (%, *w*/*w*)	Cushioning Layer (%, *w*/*w*)
Pellets	24.0	24.0	-
PH-101	62.0 [81.6]	-	86.0
SMCC	-	62.0 [81.6]	-
Polyethylene glycol 3350	7.5 [9.9]	7.5 [9.9]	7.5
SSF	0.5 [0.7]	0.5 [0.7]	0.5
Kollidon VA 64 Fine	5.0 [6.6]	5.0 [6.6]	5.0
Sodium starch glycolate	1.0 [1.3]	1.0 [1.3]	1.0

Note: The filler material consisted of the various excipients (excluding pellets). The values shown in the square brackets indicate the percent weight of the component with respect to the filler material.

**Table 2:** Tableting rates used and the corresponding dwell time and tableting output values.

Tableting Rate (rpm)	Dwell Time (ms)	Tableting Output (Tablets/h)
20	28	36,000
60	9	108,000
100	6	180,000

**Table 3:** Properties of sustained release coated pellets.

Pellet Property	AC	EC
Aspect ratio	1.11 ± 0.08	1.11 ± 0.08
Roundness	1.09 ± 0.02	1.09 ± 0.02
D_50_ (μm)	785.37	792.76
Span	0.13	0.12
True density (g/cm^3^)	1.48 ± 0.01	1.41 ± 0.00
Sugar core D_50_ (μm)	647.01	
Drug layered pellet D_50_ (μm)	760.98	
Drug layer thickness (μm)	56.99	
Coat thickness (μm)	12.20	15.89
Approximate pellet weight (g)	~0.30 (100%)	
Approximate sustained release coat weight (g) Approximate sugar core weight (g)	~0.03 (9%) ~0.16 (52%)	
Approximate drug layer weight (g)	~0.12 (39%)	
Crushing strength (MPa)	12.23 ± 1.47	11.63 ± 1.77
Elastic modulus (MPa)	148.35 ± 22.01	129.79 ± 19.35
Compression energy (mJ)	0.34 ± 0.12	0.29 ± 0.09
MDT_UC_	108.29 ± 2.42	124.51 ± 0.72

±standard deviation; values in brackets represent the relative pellet compositions by weight.

**Table 4:** Pellet volume fraction (%) values of MUPS tablets prepared using different precompression levels with different pellets and filler materials.

Precompression Level (MPa)	AC		EC	
	PH-101	SMCC	PH-101	SMCC
0	17.36 ± 0.12	16.64 ± 0.09	17.49 ± 0.10	16.84 ± 0.15
7.5	17.30 ± 0.14	16.83 ± 0.03	17.46 ± 0.07	16.93 ± 0.03
15.0	17.43 ± 0.19	16.84 ± 0.18	17.43 ± 0.05	16.80 ± 0.11

±standard deviation.

**Figure A1:**
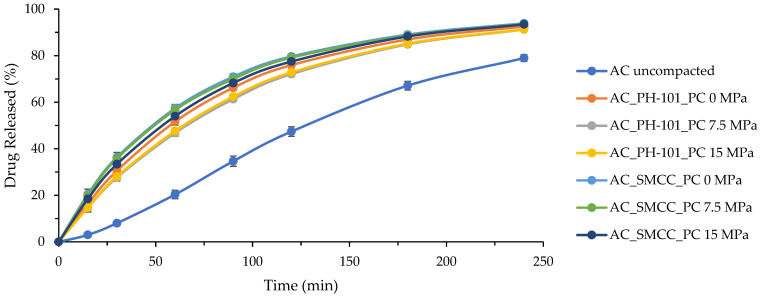
Dissolution profiles of AC pellets compacted at 40 MPa with different filler materials and using different levels of precompression (PC). Error bars represent the standard deviation. The data legend is included on the right for identification of dissolution profiles.

**Figure A2:**
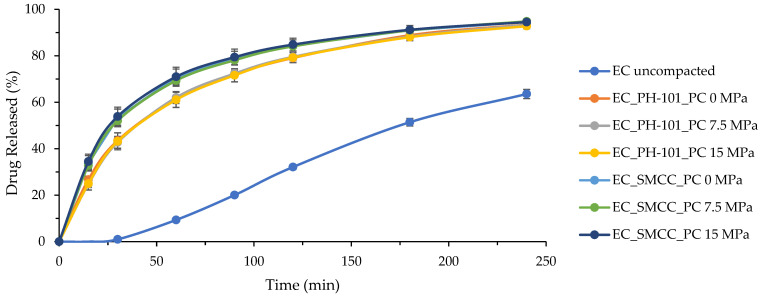
Dissolution profiles of EC pellets compacted at 40 MPa with different filler materials and using different levels of precompression (PC). Error bars represent the standard deviation. The data legend is included on the right for identification of dissolution profiles.

**Figure A3:**
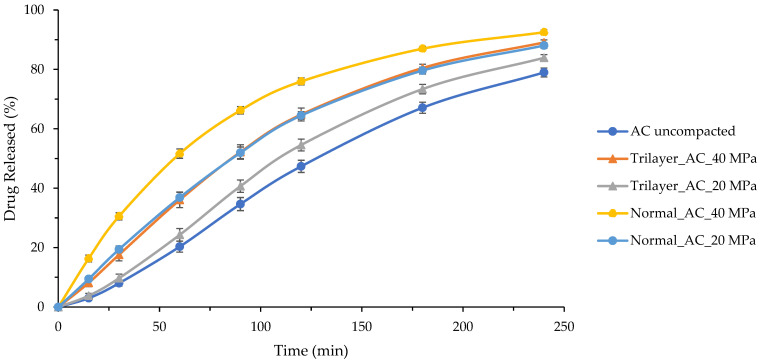
Dissolution profiles of uncompacted and compacted AC pellets. AC pellets were compacted in either normal or trilayer MUPS. Compaction was carried out using a compaction pressure of either 20 or 40 MPa. Error bars represent the standard deviation. The data legend is included on the right for identification of dissolution profiles.

**Figure A4:**
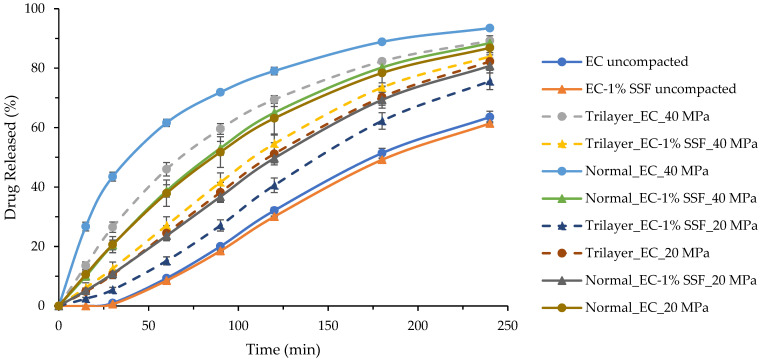
Dissolution profiles of uncompacted and compacted EC pellets. EC pellets were compacted in either normal or trilayer MUPS tablets and were used normally or lubricated with SSF. Compaction was carried out using a compaction pressure of either 20 or 40 MPa. Error bars represent the standard deviation. The data legend is included on the right for identification of dissolution profiles.

**Figure A5:**
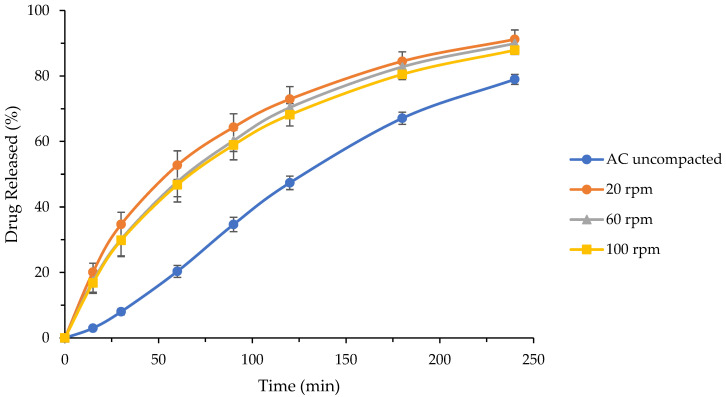
Dissolution profiles of uncompacted and compacted AC pellets prepared at various tableting rates when simulating a rotary tablet press. Error bars represent the standard deviation. The data legend is included on the right for identification of dissolution profiles.

**Figure A6:**
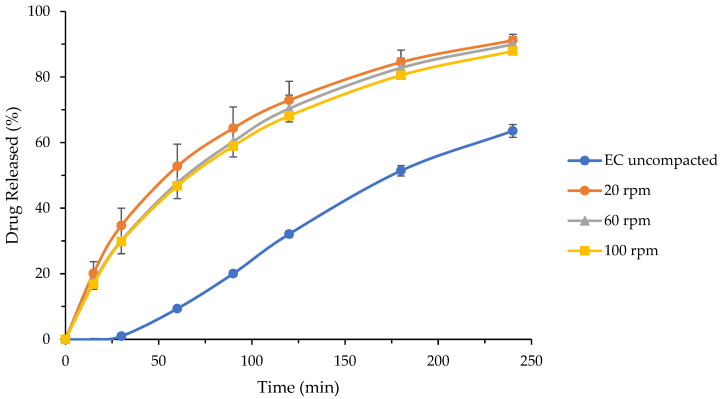
Dissolution profiles of uncompacted and compacted EC pellets prepared at various tableting rates when simulating a rotary tablet press. Error bars represent the standard deviation. The data legend is included on the right for identification of dissolution profiles.

## Data Availability

Data is contained within this article.

## Abbreviations

The following key abbreviations are used in this manuscript:

AC	Acrylic polymer
ANOVA	Analysis of variance
EC	Ethylcellulose
EC-1% SSF	Ethylcellulose coated pellets lubricated with 1%, *w*/*w* sodium stearyl fumarate
E_MDT_	Extent of pellet coat damage
*f*2	Similarity factor
MCC	Microcrystalline cellulose
MDT	Mean dissolution time
MDT_C_	Mean dissolution time of compacted pellets
MDT_UC_	Mean dissolution time of uncompacted pellets
MUPS	Multi-unit pellet system
PC	Punch displacement for precompression
SMCC	Silicified microcrystalline cellulose
SSF	Sodium stearyl fumarate
USP	United States Pharmacopeia

## Appendix A

**Table A1:** MUPS tablet volume (mm^3^) values when prepared with different precompression levels.

Precompression Level (MPa)	AC		EC	
	PH-101	SMCC	PH-101	SMCC
0	242.5 ± 1.7	252.9 ± 1.4	245.0 ± 1.4	254.5 ± 2.3
7.5	243.3 ± 2.0	250.1 ± 0.5	245.4 ± 1.0	253.1 ± 0.4
15.0	241.5 ± 2.7	250.0 ± 2.7	245.8 ± 0.6	255.1 ± 1.7

±standard deviation.
